# Editorial: Advances in plant proteomics

**DOI:** 10.3389/fpls.2022.1072217

**Published:** 2022-10-31

**Authors:** Joshua L. Heazlewood, Ian S. Wallace, Shou-Ling Xu

**Affiliations:** ^1^ School of BioSciences, University of Melbourne, Melbourne, VIC, Australia; ^2^ Department of Biochemistry and Molecular Biology, University of Nevada, Reno, Reno, NV, United States; ^3^ Department of Plant Biology, Carnegie Institution for Science, Stanford, CA, United States

**Keywords:** proteomics, plants, quantitation, mass spectrometry, methods

Numerous advances in protein mass spectrometry have been applied to plant systems over the past few decades (Jorrin-Novo, 2021). The intention of this Research Topic was to highlight some of these approaches in plants at a suitable level of detail to enable easy adoption by researchers.

The unifying feature of submissions to the Research Topic were associated with the application of protein quantitation or quantitative proteomics. Whole sample quantification of mRNA (expression profiling) has been possible since the 1990s with the development of techniques such as DNA microarray technologies (Bumgarner, 2013), and these approaches have rapidly expanded with the advent of massively parallel sequencing technologies. By contrast, quantification of whole-proteome dynamics (e.g., cell, organ or tissue) is still not possible, even with the considerable technical advances in mass spectrometry that have occurred in the past 20 years (Timp and Timp, 2020). Nonetheless, quantitative proteomic approaches are a major driver for many plant biologists engaging with protein mass spectrometry to gain functional proteome or sub-proteome insights into the dynamics of protein abundance in plant systems (Mergner and Kuster, 2022).

The resolving power of mass spectrometers for proteomics enabled quantitative approaches that incorporate isotopic labelling with stable heavy isotopes such as ^15^N (Schulze and Usadel, 2010). Since these instruments can discriminate between heavy (^15^N) and light (^14^N) peptides, these metabolically labelled samples can be analysed in a single run e.g., control (light) and treatment (heavy). Such an approach considerably reduces run to run variation and enables reliable protein quantification. This Research Topic illustrates two methods that highlight the advantages of ^15^N labelling for quantitative proteomics. Shrestha et al. presents a data analysis workflow outlining the steps used to extract quantitative information from a ^15^N labelled analysis. Reyes et al. outlines the application of targeted proteomics or parallel reaction monitoring (PRM) to ^15^N labelled samples highlighting potential advantages of combining both labelling and PRM for reliable quantification.

Metabolic labelling with stable heavy isotopes necessitates near complete incorporation of the heavy isotope label, and deviations from this situation result in more complex data matrices and complications in downstream analyses (Schulze and Usadel, 2010). Metabolic labelling with stable heavy isotopes is done at the protein level during plant growth, and thus this application is restricted to certain plant species that can achieve high incorporation for desirable quantitative results; two samples can be mixed and measured in a single run. Alternative labelling methods, such as chemical tagging with isobaric tags (iTRAQ, TMT) were developed to label samples at the peptide level and to facilitate sample multiplexing in a single run (Dayon and Affolter, 2020). Li et al. have demonstrated the power of this approach to explore changes in the root proteome of tobacco exposed to different soil types. The labelling method has enabled three samples to be analysed in a single run and then absolute and relative protein changes measured. Zhao et al. has also embraced the iTRAQ labelling approach in a quantitative survey of the rice leaf proteome and its response to rice blast fungal infection over time. In this experimental setup, three biological replicates at both 0 hour and 24 h could be analysed and quantified by tandem mass spectrometry in a single run. This again highlights the utility of this approach with its integration in a standard experimental workflow and the ability to multiplex samples. With the TMTpro 18 kit, up to 18 samples can be measured simultaneously in a single run (Li et al., 2021).

The application of quantitative proteomics in plant biology has kept pace with other disciplines in embracing and developing advances in protein mass spectrometry. Whole sample protein quantitation remains one of this field’s main objectives, but the majority of methods presented above are technically limited by the fact that they are Data Dependent Acquisition (DDA) methods, which will preferentially quantify the most abundant proteins in a sample first, thereby limiting the proteomic depth that can be analysed in a given sample. In this light, we are excited by the potential of Data Independent Acquisition (DIA) methods that hold promise for more rigorous quantification of proteins across a large dynamic range of protein abundances. Additionally, we suggest that the field considers new computational tools to aggregate and organize quantitative proteomic datasets as they become available. This goal is certainly challenging based on the wide variety of quantitative experiments that can be performed and the range of instruments that can be utilized, but in analogy to transcriptomic experiments, we see considerable value in the aggregation of quantitative proteomics data for meta-analysis.

## Author contributions

JLH assembled the initial draft. ISW and SLX extended and refined the text. All authors contributed to the article and approved the submitted version.

## Funding

This work was funded by the NIH grant R01GM135706 and S10OD030441 to SLX.

## Acknowledgments

We would like to thank all authors for their contributions to this Research Topic. Furthermore, we are grateful to all reviewers and editors for their help in evaluating all manuscripts and the editorial office for their support.

## Conflict of interest

The authors declare that the research was conducted in the absence of any commercial or financial relationships that could be construed as a potential conflict of interest.

## Publisher’s note

All claims expressed in this article are solely those of the authors and do not necessarily represent those of their affiliated organizations, or those of the publisher, the editors and the reviewers. Any product that may be evaluated in this article, or claim that may be made by its manufacturer, is not guaranteed or endorsed by the publisher.

## References

[B1] Bumgarner R. (2013). Overview of DNA microarrays: types, applications, and their future. Curr. Protoc. Mol. Biol. 101, 22.21.21–22.21.11. doi: 10.1002/0471142727.mb2201s101 PMC4011503 23288464

[B2] Dayon L. Affolter M. (2020). Progress and pitfalls of using isobaric mass tags for proteome profiling. Expert Rev. Proteom. 17, 149–161. doi: 10.1080/14789450.2020.1731309 32067523

[B3] Jorrin-Novo J. V. (2021). Proteomics and plant biology: Contributions to date and a look towards the next decade. Expert Rev. Proteom. 18, 93–103. doi: 10.1080/14789450.2021.1910028 33770454

[B4] Li J. Cai Z. Bomgarden R. D. Pike I. Kuhn K. Rogers J. C. . (2021). TMTpro-18plex: The expanded and complete set of TMTpro eeagents for sample multiplexing. J. Proteome Res. 20, 2964–2972. doi: 10.1021/acs.jproteome.1c00168 33900084 PMC8210943

[B5] Mergner J. Kuster B. (2022). Plant proteome dynamics. Annu. Rev. Plant Biol. 73, 67–69. doi: 10.1146/annurev-arplant-102620-031308 35138880

[B6] Schulze W. X. Usadel B. (2010). Quantitation in mass-spectrometry-based proteomics. Annu. Rev. Plant Biol. 61, 491–516. doi: 10.1146/annurev-arplant-042809-112132 20192741

[B7] Timp W. Timp G. (2020). Beyond mass spectrometry, the next step in proteomics. Sci. Adv. 6, eaax8978. doi: 10.1126/sciadv.aax8978 31950079 PMC6954058

